# Punicic Acid and Its Role in the Prevention of Neurological Disorders: A Review

**DOI:** 10.3390/foods11030252

**Published:** 2022-01-18

**Authors:** Claudia M. Guerra-Vázquez, Mariana Martínez-Ávila, Daniel Guajardo-Flores, Marilena Antunes-Ricardo

**Affiliations:** Tecnológico de Monterrey, Centro de Biotecnología-FEMSA, Escuela de Ingeniería y Ciencias, Av. Eugenio Garza Sada 2501 Sur, Monterrey C.P. 64849, NL, Mexico; a00832550@itesm.mx (C.M.G.-V.); mm.avila@tec.mx (M.M.-Á.); danielgdo@tec.mx (D.G.-F.)

**Keywords:** antioxidant, conjugated linoleic acid, blood–brain barrier, Alzheimer’s disease, Parkinson’s disease, Huntington’s disease, neurodegeneration

## Abstract

Millions of people worldwide are affected by neurodegenerative diseases (NDs). NDs are characterized by progressive damage and death of nerve cells accompanied by high levels of inflammatory biomarkers and oxidative stress conditions. Punicic acid, the main bioactive component of pomegranate (*Punica granatum*) seed oil, is an omega-5 isomer of conjugated α-linoleic acid that has shown strong anti-oxidative and anti-inflammatory effects that contributes towards its positive effect against a wide arrange of diseases. Punicic acid decreases oxidative damage and inflammation by increasing the expression of peroxisome proliferator-activated receptors. In addition, it can reduce beta-amyloid deposits formation and tau hyperphosphorylation by increasing the expression of GLUT4 protein and the inhibition of calpain hyperactivation. Microencapsulated pomegranate, with high levels of punicic acid, increases antioxidant PON1 activity in HDL. Likewise, encapsulated pomegranate formulations with high levels of punicic acid have shown an increase in the antioxidant PON1 activity in HDL. Because of the limited brain permeability of punicic acid, diverse delivery formulations have been developed to enhance the biological activity of punicic acid in the brain, diminishing neurological disorders symptoms. Punicic acid is an important nutraceutical compound in the prevention and treatment of neurodegenerative diseases such as Alzheimer’s, Parkinson’s, and Huntington’s disease.

## 1. Introduction

Some of the most prevalent diseases that can cause loss of independence in older populations are neurodegenerative diseases (NDs), which are becoming more frequent. The neurodegenerative process is the progressive loss of function or death of central nervous system cells, causing an increase in motor and cognitive impairments with time [1]. Among the most prevalent NDs are Alzheimer’s Disease (AD) and frontotemporal dementia, Parkinson’s Disease (PD), Huntington’s Disease (HD), Amyotrophic Lateral Sclerosis (ALS), and multiple spinocerebellar ataxias. AD incidence in the population aged 85 and over is about 30%, while PD is around 2% in people above 65 years old, and ALS reported 1–2 cases per 100,000 people yearly, and the incidence is expected to soar as the population ages [2]. Therefore, there is a need for the implementation of new preventive measures and the development of novel treatments for the early stages of neurodegeneration. The World Health Organization estimates that the global social cost of dementia is USD 818 billion, equivalent to 1.1% of the world’s gross domestic product. The prevalence of AD in Latin America is as high as 8.5%. Moreover, it is expected that by 2030 about 65.7 million will live with dementia and around 115.4 million by 2050 [3]. The mortality and people’s disability caused by these neurological disorders has increased, hence, considering them as a global public health challenge. As the incidence is expected to soar as the population ages, finding new solutions and strategies for the treatment of neurodegenerative diseases is a goal of increasing urgency. Because oxidative damage and inflammation are key pathways in the development of neurodegeneration, phytochemicals with elevated anti-oxidative and anti-inflammatory properties are being investigated to aid in the prevention of neurodegeneration and halt disease progression.

Pomegranate (*Punica granatum*) is an ancient and adaptable fruit original from Western Asia that belongs to the Punicaceae family. It is cultivated throughout the world, including Middle Eastern, Asian, European, and American countries, mainly in subtropical and tropical areas under variable climatic conditions [4,5]. Approximately 50% of the total weight of the fruit corresponds to the peel, which is an important source of phenolic compounds, minerals, and complex polysaccharides. Meanwhile, the edible part of the pomegranate fruit consists of arils (40%) rich in water, sugars, pectin, and seeds (10%) [6]. Pomegranate seeds contain many components such as polyphenols and fatty acids that contribute to their beneficial effects. Pomegranate Seed Oil (PSO) represents around 12% and 20% of the total seed weight [7]. PSO contains 14 fatty acids, the most abundant of which is punicic acid 50–80% [7,8,9], followed by linoleic acid (13–20%), palmitic acid (6–9%), stearic acid (2–3%), oleic acid (8–9%), linolenic acid (0.06–0.08%), and arachidic acid (0.68–0.90%) [9]. Punicic acid, PSO’s main bioactive component, was shown to achieve a potent anti-oxidative effect that contributes towards its positive effect against a wide arrange of diseases such as osteoporosis, has anti-obesity properties, increases the expression of antioxidant and lipid metabolism-related genes, and modifies the composition and function of high-density lipoprotein (HDL) [10,11,12,13].

Punicic acid is an omega-5 isomer of conjugated α-linolenic acid (CLnA) and exhibits structural similarities to conjugated linoleic acid (CLA) [12]. By itself, punicic acid possesses a wide spectrum of biological effects such as anti-inflammatory, anti-diabetic, anti-obesity, anti-proliferative, and anti-carcinogenic properties [14,15]. The main biological mechanism described for punicic acid involves the modulation of the differential expression of peroxisome proliferator-activated receptors (PPARs), which control the expression of genes involved in cell differentiation and proliferation, regulate enzymes involved in lipids metabolism, and glucose homeostasis. In addition, PPARs are closely related to the activation and production of pro-inflammatory biomarkers [16,17,18,19]. While the antioxidant and anti-inflammatory properties of punicic acid may provide beneficial effects on the treatment of NDs, the way it interacts in different pathways related to the progression of NDs may give it advantages over other anti-oxidative nutraceuticals. This review aimed to present an overview of the current knowledge about the potential benefits of punicic acid in neurological disorders and the molecular mechanism involved in its effects.

## 2. Main Pathways Involved in Neurological Disease

**Figure 1:**
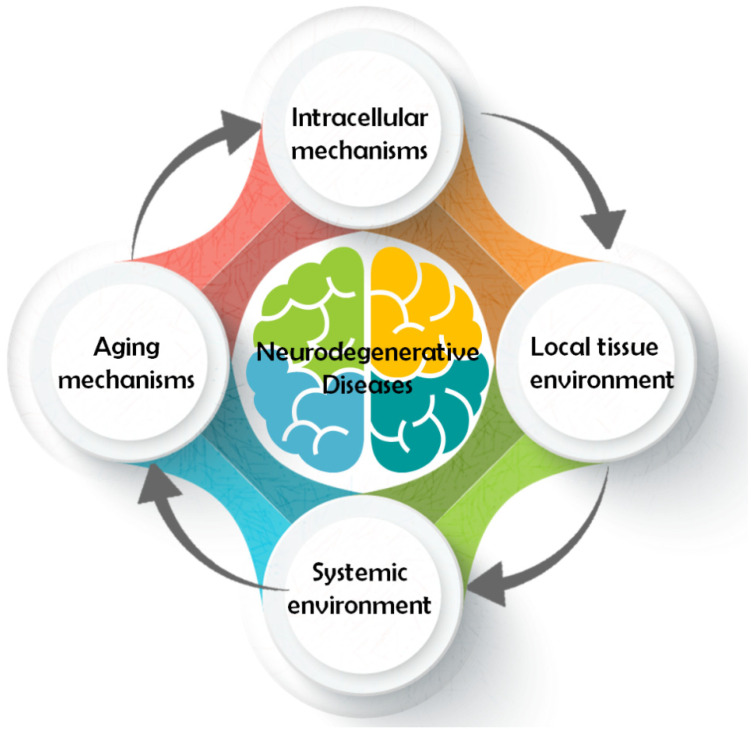
Classification of the pathways involved in neurodegenerative diseases (NDs).

**Figure 2:**
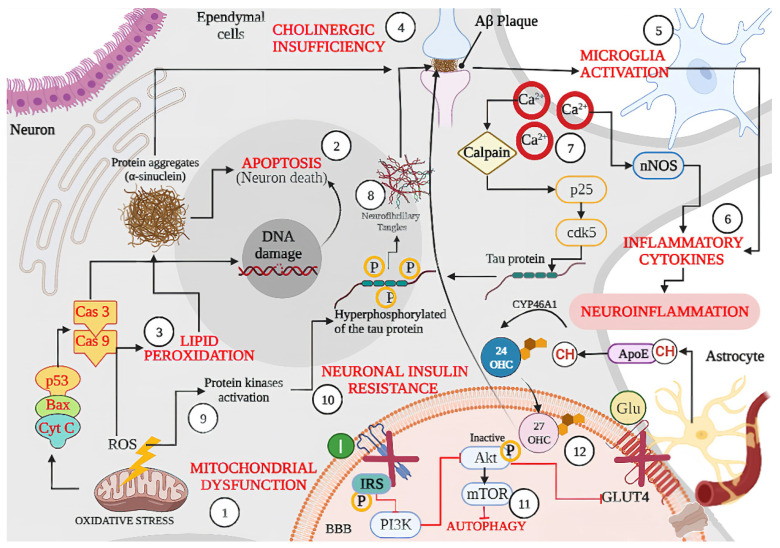
Schematic representation of shared physiopathological hallmarks in neurodegenerative diseases (NDs): (1) Mitochondrial dysfunction due to oxidative stress, aging, or because of genetic or environmental factors damage, resulting in the excessive production of ROS, which can activate p53 and the Bax (apoptotic regulator) translocation that allows the release of cytochrome C (Cyt C) leading the (Cas 9) and caspase 3 (Cas3) activation, resulting in DNA damage and cell death or (2) Apoptosis. Likewise, excessive ROS production also leads to oxidative stress and (3) Lipid Peroxidation, which can lead to protein aggregates such as α-synuclein as well as misfolded amyloid β peptide, the latter becoming an amyloid β (Aβ) plaque affecting neuron signaling induced by (4) Cholinergic Insufficiency. In turn, accumulation of Aβ plaque induces (5) Microglia Activation with the concomitant release of (6) Inflammatory Cytokines and produces neuroinflammation. On the other hand, (7) Dysregulation of Ca^2+^ because of neuronal membrane depolarization could induce synaptic deficits and promote the accumulation of Aβ plaques, and (8) Neurofibrillary Tangles through calpain activation. In addition, sustained calcium inflow results in over-activation of neuronal nitric oxide synthase (nNOS), with the increase in nitric oxide synthesis leading to oxidative stress/nitrosative stress and generalized brain inflammation. Moreover, ROS accumulation induces (9) kinases activation (glycogen synthase kinase-3β, GSK-3β) and induces tau hyperphosphorylation, promoting the accumulation of Aβ plaques. Accumulation of Aβ oligomers causes removal of insulin receptors (IRS) from the cell surface, inducing a (10) Neuronal Insulin Resistance and inhibiting the activation of glucose transporter type 4 (GLUT 4). Dysfunctional insulin signaling brings mammalian target of rapamycin (mTOR) pathway down and results in (11) Autophagy failure to accumulate Aβ plaques. Finally, the synthesized cholesterol binds apolipoprotein E (APOE) to form APOE–cholesterol (APOE–CH) particles. APOE–CH particles are internalized into neurons, and the free cholesterol is metabolized to 24-hydroxycholesterol (24-OHC), which subsequently passes through the blood–brain barrier (BBB) and enters into plasma, while plasma (12) 27 hydroxylcholesterol (27-OHC) flows into the brain, increasing the level of α-synuclein and eventually forms Lewy bodies (LBs). Back lines indicate stimulation, while red lines indicate inhibition.

Even though all ND have different pathology and symptomatology, their pathways share some common traits. A conceptual model classifying the different pathways involved in neurodegeneration was developed considering four major models of action [20] (Figure 1). In general, pathways that contribute to neuron survival and degeneration include: (1) intracellular mechanisms such as apoptosis [21], autophagy [22], mitochondrial function, oxidative damage and repair [23], ubiquitin/proteasome [24], (2) local tissue environment such as cell adhesion [25], endocytosis, neurotransmission [26], prions/transmissible factor [27], (3) systemic environment such as inflammation/immune response [28], lipid/endocrine metabolism [29], brain vasculature [30], (4) and mechanisms related to aging [31], for instance epigenetics [32], neurotrophic factors [33], and telomeres [34]. All these components are highly related and interact with each other to modulate the neurodegenerative process (Figure 2).

### 2.1. Intracellular Mechanism

Among intracellular mechanisms related to neuron survival and degeneration, DNA damage and defective repair are the most common hallmarks that many ND’s with features of progressive movement disorders share. A high concentration of reactive oxygen species (ROS) can cause accumulation of oxidative DNA damage in its sequence and epigenetic modifications [24]. Altered gene expression could cause loss of normal neural function and progressively trigger programmed cell death and neuronal loss [22]. Mitochondria is the major source of cellular ROS production, and it was found that oxidative damage can promote α-synuclein aggregation and affect amyloid-β (Aβ) and other proteins related to aging and ND [22,35].

In long-living, non-mitotic cells such as neurons, ROS abundance causes oxidative stress and impairment of antioxidant defenses, resulting in dysfunction of the mitochondria and initiation of cell death cascade [36]. Multiple studies relate the effects of nitric oxide and ROS with NDs, including nitration of Lewis bodies in Lewis body dementia and Alzheimer’s Disease (AD), nitration of α-synucleins in patients with multiple system atrophy, widespread nitrates tau proteins in AD, and frontotemporal dementia with Parkinsonism. Decreased levels of nitric oxide contribute to the upregulation of Aβ in the cerebrovascular system, and nitric oxide inhibition delays the progression of Parkinson’s Disease pathology [37]. Likewise, Tumor Necrosis Factor-alpha (TNF-α) is a pro-inflammatory cytokine related to the pathogenesis of ND through systemic inflammation [38]. Anti-TNF-α therapies were proposed by several studies to diminish AD pathology, decreasing amyloid deposition and diminishing neuronal impairment [39].

In addition, brain insulin resistance was described as a factor to induce cognitive impairments and neurodegeneration. Insulin brain levels are reduced during aging and Alzheimer resulting in the inhibition of several phosphatases involved in Tau dephosphorylation resulting in the deposition and accumulation of extracellular amyloid-β (Aβ) plaques [40,41].

### 2.2. Local Tissue Environment

The progressive aggregation of misfolded proteins that severely affect the local tissue environment, creating damage, is a pathological feature that characterizes neurodegenerative diseases [42]. These misfolded proteins are subjected to protein degradation, such as proteasome-mediated. Inhibition of protein degradation pathways leads to the formation of protease-resistant, thus, decreasing the propagation of aggregated proteins that promote the misfolding of cell proteins [43]. Likewise, autophagy is the main mechanism responsible for removing protein aggregates, dysfunctional cellular organelles, and pathogens to maintain cellular homeostasis. Accumulation of immature autophagic vacuoles (AVs) as a consequence of a disrupted autophagy process is a common characteristic observed in the brain of Alzheimer’s patients. It was shown that mammalian target of rapamycin (mTOR) signaling is inhibited in the cortex and hippocampus of adult AD model mice. Brain insulin resistance induces alterations in the insulin/insulin-like growth factor (IGF-1)-PI3K (phosphoinositide 3-kinase class I)-Akt pathway, resulting in the aberrant activation of mTOR signaling, which negatively regulates autophagy induction [44,45,46].

### 2.3. Systemic Environment

Changes in the systemic environment such as inflammation are common in neurodegenerative diseases such as AD and Parkinson’s Disease (PD) and can cause, along with oxidative stress, perturbances in the proteome composition of High-Density Lipoprotein (HDL) [47]. Circulating HDL provides resilience to cerebrovascular dysfunction in AD, which plays an important role in brain metabolism and homeostasis, dampening the clearance of Aβ and tau and thus leading to the formation of neuritic plaques and neurofibrillary tangles [48].

### 2.4. Aging Mechanism

The composition of fatty acids and fluidity of brain membranes change with age. Polyunsaturated Fatty Acids (PUFAs) such as docosahexaenoic acid (DHA, 22:6 n-3) and arachidonic acid (AA, 20:4 n-6) are the most abundant and important PUFAs in the brain and play a critical role in aging and neurodegeneration. In the elderly, DHA and AA decrease in membranes of the orbitofrontal cortex. Specific DHA deficiency might be caused by an age-related reduction in enzyme activity involved in the regulation of DHA synthesis, uptake, and assembly into brain phospholipids (Zhang et al., 2018). Meanwhile, high dietary consumption of omega-3 and omega-6 PUFAs is favorable for the memory of healthy older human adults. This process is mediated by the integrity and preservation of the white matter microstructure of the fornix in the brain (Zamroziewicz et al., 2017).

Several PUFAs such as DHA and AA are being studied for the development of new treatments against NDs and neurodegeneration [49,50]. Punicic acid (18:3, Δ9cis, 11trans, 13cis, n-5) is a promising candidate whose mechanism of action is yet to be completely understood. The following section will refer to the characteristics and mechanisms of interest of punicic acid and their potential relation with the prevention of NDs.

## 3. Punicic Acid

**Figure 3:**
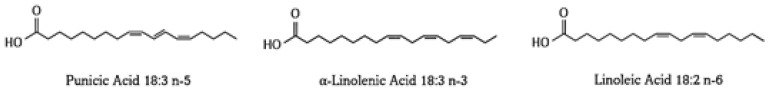
Structure of punicic acid and related isomers α-linolenic acid and linoleic acid. Chemical structures drawn in ChemDraw.

In nature, the most abundant source of punicic acid (PuA) is pomegranate (*Punica granatum*), with the final amount depending on the fruit genotype. However, other sources include *Momordica balsamina*, *Ecballium elaterium*, *Fevillea trilobata*, and some species from the *Trichosanthes* genus, such as *T. kirilowii*, *T. anguina*, *T. bracteata*, *T. nervifolia* [14,19,51]. Punicic acid, also known as octadecatrienoic acid or trichosanic acid (C_18_H_30_O_2_), possesses a molecular weight of 278.43 g/moL, a melting point of 44–45 °C, and an octanol–water partition coefficient (X LogP) of 6.4. Moreover, it was reported that punicic acid has a molar refractivity and polarizability value of 89.64 m³/moL and 35.91 Å³, respectively [52]. PA exhibits a pKa value of 4.99 (strongest acidic), as it is able to act as a donator of one hydrogen [53,54]. It is a conjugated linolenic acid isomer with structural similarities to α-linolenic and linoleic acids [54] (Figure 3). Among the main described characteristics of punicic acid is its ability to scavenge hydroxyls, metal chelation, and reduce properties [15].

**Figure 4:**
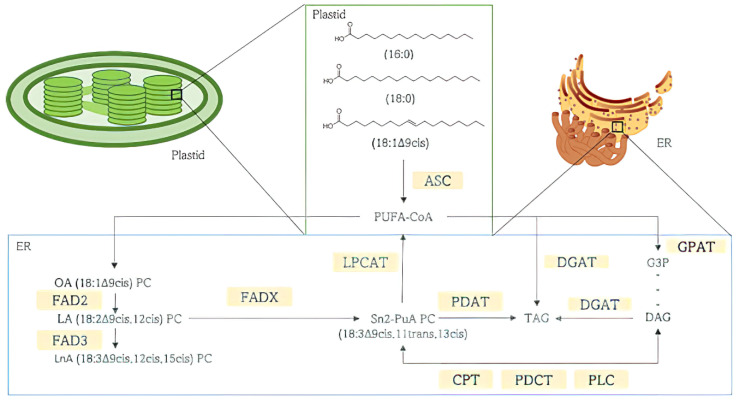
Punicic acid (PuA) biosynthesis and storage in triacylglycerol (TAG). Phosphatidylcholine (PC), Oleic acid (OA), Linoleic acid (LA), position sn2 Punicic Acid (PuA), Punicic Acid Phosphatidylcholine (Sn2-PuA PC), Fatty Acid Desaturase (FAD) 2 and FADXs, acyl-Coenzyme A (CoA), Acyl-CoA synthetase (ACS), Triacylglycerol (TAG) Phospholipid:diacylglycerol Acyltransferase (PDAT), Lysophosphatidylcholine Acyltransferase (LPCAT), n-glycerol-3-phosphate acyltransferase (GPAT), diacylglycerol acyltransferase (DGAT), sn-glycerol-3-phosphate (G3P), sn1,2-diacylglycerol (DAG), CDP-choline:1,2-diacyl-sn-glycerol cholinephosphotransferase (CPT), phosphatidylcholine: diacylglycerol cholinephosphotransferase (PDCT), phospholipase C (PLC).

Biosynthesis of punicic acid begins with the de novo synthesis of fatty acids inside the plant’s plastid, mostly palmitic (16:0), stearic (18:0), and oleic acids (18:1Δ9cis) (Figure 4). Fatty acids are conjugated on phosphatidylcholine (PC) to undergo desaturations and conjugations in the position sn2 of PC. Oleic acid-PC (OA 18:1Δ9cis) is processed into linoleic acid-PC (LA 18:2Δ9cis,12cis), which in turn is transformed into Punicic Acid-PC by fatty acid desaturase (FAD) 2 and fatty acid desaturases group X (FADXs), respectively. Newly synthetized fatty acids are then converted into acyl-Coenzyme A by the action of Acyl-CoA synthetase to act as acyl donors in triacylglycerol (TAG) biosynthesis inside the endoplasmic reticulum (ER) before being stored in cytoplasmic lipid droplets [19,51,54].

The main challenges of using PSO or punicic acid for health applications are chemical instability and limited water solubility [55]. Since fatty acids such as punicic acid are molecules highly unsaturated, they are susceptible to degradation due to oxidation, light, or thermal treatments. Likewise, because punicic acid is poorly soluble in water and only a small fraction can be slowly absorbed by the body, the bioavailability of this molecule is very low and exhibits a rapid metabolism to conjugated linoleic acid (CLA), limiting its use in commercial or therapeutic [56]. In order to overcome these challenges, researchers have explored different strategies such as the synthesis of precursor molecules and the design of specific delivery systems to protect the active drug. Modifications on the chemical structure can protect the molecule’s active sites from biological degradation and therefore improve its stability. Esterification of punicic acid showed an improvement by 30% in the oxidative stability of monodispersed punicic acid compared to its free form. Likewise, this chemical modification of punicic acid significantly improved its water solubility and bioaccessibility [55]. On the other hand, encapsulation is the most used technique to protect drugs from environmental and chemical degradation. In this sense, spray-drying microencapsulation of pomegranate seed oil using succinylated taro starch demonstrated 61% of encapsulation efficiency with an improvement in the oxidation stability and a significant delivery of PSO in the small intestine [57]. Likewise, PSO nanoemulsions have shown improved stability under stress conditions such as osmotic stress and extreme pH values [58]. Mizrahi et al. [59] reported that nanoemulsions of pomegranate seed oil exhibited strong neuroprotective effects by reducing lipid oxidation and neuronal loss.

Recent technological advances allowed the development of novel delivery systems, which not only protect the drug but also exhibit an efficient release in the target site, improving the bioavailability and biological activity of this by the modification of the pharmacokinetics parameters [60,61]. Improved physical and peroxidation stability of PSO at different temperatures (4 °C and 25 °C) was achieved by the incorporation of beeswax and propolis wax during the fabrication of PSO nanostructured lipid carriers. After 40 days of storage time, PSO nanostructured lipid carriers at 4 °C showed peroxidation levels significantly lower than at 25 °C. Likewise, the antioxidant activity of these systems, measured by DPPH free radical-scavenging activity, showed to be stable throughout the storage period regardless of temperature conditions [62]. Moreover, the combination of PSO with other therapeutic drugs or nutraceuticals was shown to improve the pharmacokinetic parameters and biodistribution profile of the latter [63,64]. The biological benefits of PSO and punicic acid also attracted the food industry’s interest to design and offer consumers more healthy products through the enrichment in polyunsaturated acids of the different food matrices [65,66,67,68].

These approaches can be really useful in the development of food products with an enhanced nutritional quality or even for the development of food supplements that contribute to preserving human health [69].

### 3.1. Punicic Acid Metabolism

Lipids are abundant in the brain, and they perform a variety of structural functions such as neurogenesis, signal transduction, neural communication, membrane compartmentalization, synaptic transmission, and regulation of gene expression [70]. Punicic acid (PuA) is metabolized into circulating conjugated linoleic acid CLA via a saturation reaction [71,72,73,74].

**Figure 5:**
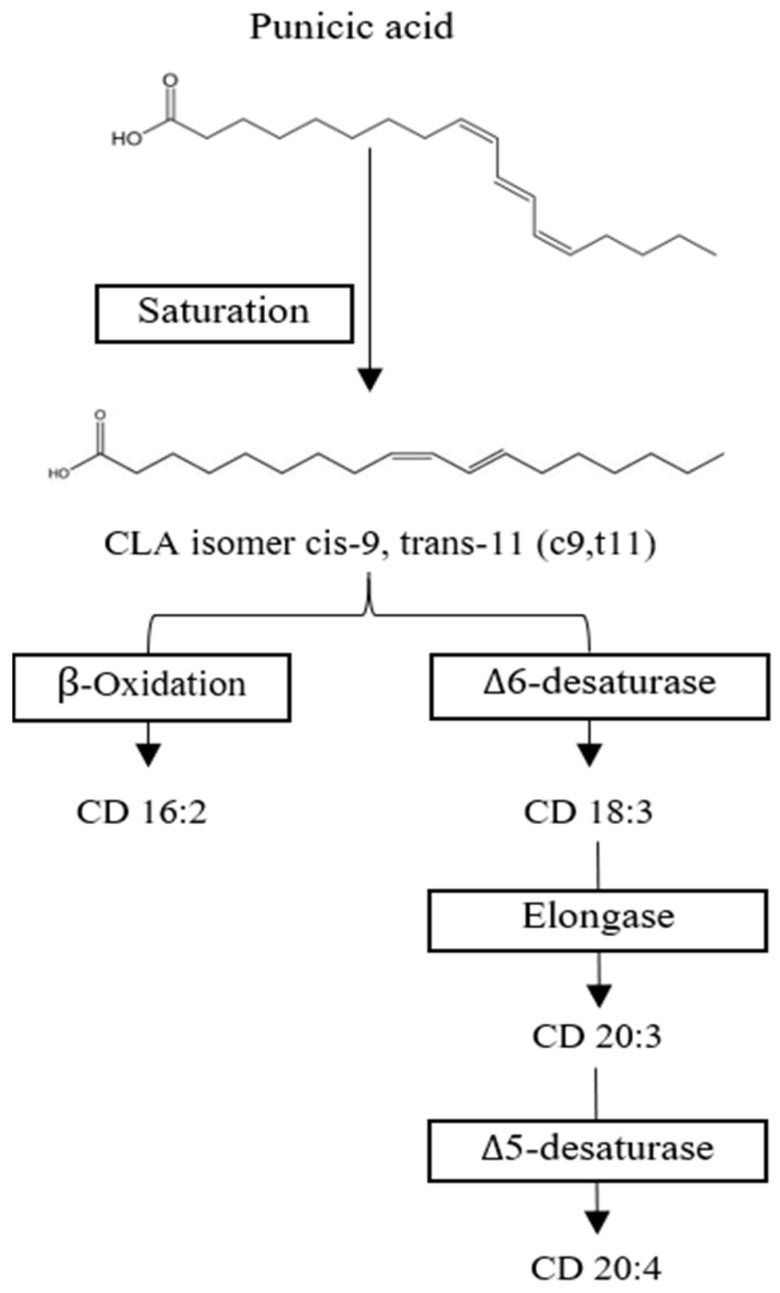
Proposed punicic acid metabolism. Punicic acid is transformed into conjugated linoleic acid (CLA cis-9, trans-11) and then either β-oxidized into Conjugated Diane (CD) 16:2 or metabolized by Δ6-desaturase to become CD 18:3 to be further processed into CD 20:3 and CD 20:4. Chemical structures drawn in ChemDraw.

CLA is mostly processed in the liver into neutral lipids and phospholipids, respectively. CLA isomers c9,t11 and t10,c12 are metabolized via desaturation and elongation reactions while maintaining their conjugated diene structure [75]. Both isomers are processed differently; t10,c12 CLA is readily beta oxidized to Conjugated Diane (CD) 16:2 and delta 6 desaturated to CD 18:3, while c9,t11 CLA appears to be metabolized into CD 20:3 (Figure 5). CD 18:3, 20:3, and 20:4 are mainly incorporated into phospholipids CLA. At the same time, CD 18:3 and CD 20:3 are distributed into neutral lipids [76]. In humans, it was observed that punicic acid is transformed into c9,t11 and incorporated into tissues such as plasma, red blood cell mass, and be partially beta-oxidation in peroxisomes to produce CD 16:2 [72,75]. In rats, CLA was measured mainly in the liver, kidney, adipose tissue, mammary tissue, plasma, heart, and brain, with only small traces of punicic acid being found in liver and heart tissue [71,76]. A study measured the concentration of CLA in human plasma after daily intake of 0.8 g, 1.6 g, or 3.2 g of c9,t11 CLA in capsules and found that the metabolites CD 18:3 and 16:2 were promptly incorporated in a linear fashion, while 20:3 reached a plateau at 1.6 g/d [75].

A study in rats demonstrated that after 40 days of rich in punicic acid supplementation with PSO rich in punicic acid at concentrations of 1%, 2%, and 4% CLA was found in serum, liver, heart, and kidney, respectively, and some traces of PuA were found in the liver and heart. In the brain, PSO consumption was shown to decrease thiobarbituric acid reactive substances (TBARS) levels, which are used to determine lipid peroxidation, but neither PuA nor CLA was detected in this tissue [71]. However, other studies confirmed the presence and metabolism of CLA in the brain of both rats and humans [76,77,78]. CLA metabolites may be able to reach the brain through incorporation into very-low-density lipoprotein (VLDL) [76], produced by the intestine and liver, and be absorbed into the brain by the very-low-density lipoprotein receptor (VLDLR) [79]. However, it is also likely that low-density lipoprotein (LDL) and the low-density lipoprotein receptor (LDLR), as well as the fatty acid translocase (FAT/CD36), are involved in the transport of CLA through the blood–brain barrier (BBB), as it is the case with most PUFAs [80,81].

Astrocytes and endothelial cells, two major components of the BBB, are the major contributors to the transportation of PUFAs from the circulation to the brain [82]. Astrocytes participate in the synthesis of eicosanoids [76] and play an important role in CLA metabolism [79]. CLA isomers c9,t11 and t10,c12 are effectively incorporated and metabolized in rat brain and human astrocyte cell culture. However, because beta-oxidation of CLA is more efficient in the brain than in other tissues, CLA concentrations in the brain are low. Therefore, it is believed that the incorporation of CLAs is tissue-specific [76]. Low CLA concentrations in the brain could be the result of the preference of the cerebral tissue for other fatty acids, against the selection of fatty acids with *trans* double bonds, or the presence of the blood–brain barrier, poor incorporation of phospholipids, and low supply. Additionally, the incorporation of CLA in the brain is lower than in other tissues [71,76].

### 3.2. Punicic Acid Effects on Neurodegenerative Disease

Punicic acid could be related to neurodegeneration prevention through several different pathways, including (1) intracellular mechanisms related to oxidative damage through peroxisome proliferator-activated receptor (PPAR)s and high-density lipoprotein (HDL) associated paraoxonase 1 (PON1); (2) local tissue environment such as synaptic function via calpains, and (3) systemic environment such as inflammation and lipid metabolism via PPARs and glucose metabolism with glucose transporter type 4 (GLUT4) (Table 1). Punicic acid can act as an agonist of PPARγ, increasing mRNA expression of PPAR-α, PPAR-β, PPAR-γ, and PPAR- γ, and bind to both PPAR- γ and PPAR-α [83,84]. It increases GLUT4 protein expression [85] and increases the anti-oxidative properties of HDL and PON1 activity [86,87]. Finally, punicic acid can act as an inhibitor of calpain, which plays a key role in the ROS generation, and calpain may play a role in mitochondrial ROS generation and HDL degradation [88].

#### 3.2.1. Punicic Acid Increases Expression of Peroxisome Proliferators Activated Receptors (PPARs)

There is a relationship between the role of PPARs such as PPAR-α, PPAR-β/δ, and PPAR-γ and neurodegenerative disease, particularly Alzheimer. Inside the brain, activities attributed to PPAR-α include the reduction in oxidative stress, neuroinflammation, tau hyperphosphorylation, less Aβ formation and aggregation, glucose metabolism, autophagy, neurotransmission, and aspects of lipid metabolism such as fatty acyl-CoA β-oxidation and PUFA biosynthesis. Similarly, PPAR-β/δ regulates the central nervous system myelination process, while PPAR-γ is involved in neuron biogenesis, neuroinflammation, and neurodegeneration [89,90]. In patients with neurological diseases, PPARs are down-regulated [91].

The effects of punicic acid over PPARs have been studied over time. The evidence shows that punicic acid decreases inflammation induced by pro-inflammatory cytokines Tumor Necrosis Factor Alpha (TNF-α) and Interleukin 6 (IL-6) on 3T3-L1 pre-adipocytes. Likewise, punicic acid-enhanced protein expression of PPAR-γ abates transcriptional activity of Nuclear Factor Kappa B (NFκB) p65 subunit, reduced mRNA expression of suppressor of cytokine signaling 3 (SOCS3), and attenuates protein tyrosine phosphatase 1B (PTP1B) induced by TNF-α [83,84]. A more recent study in mice liver fed a high-fat diet supplemented with PSO nanoemulsions found that punicic acid increased the expression of lipid metabolism-related genes PPAR-α, PPAR-β and PPAR-γ, fatty acid synthase (Fasn), and sterol regulatory element-binding transcription factor (Srbp1), along with antioxidant genes (aldehyde oxidase 1 (Aox1), glutathione S-transferase A4 (Gst4), NAD(P)H quinone dehydrogenase 1 (Nqo1), Nrf2, and peroxiredoxin 1 (Prdx1), and decreased levels of IL-6 and TNF-α [12]. The Punicic acid effect over PPARs is also related to HDL metabolism. Rabbits supplemented with microencapsulated pomegranate showed modified lipid composition of HDL particles. PPARα and PPARγ are able to remodel HDL structure through the regulation of the expression of genes related to HDL metabolism [86].

#### 3.2.2. Punicic Acid Participation in Calpain Hyperactivation Inhibition

Calpains are calcium-dependent cysteine proteases that have been implicated in several neurodegenerative diseases such as Alzheimer’s and Huntington’s Disease. Calpains are important for synaptic function and neuroplasticity, as they exert a neuroprotective effect at base expression, while overactivation leads to neurotoxicity. Calpain-1 and calpain-2 are abundant in the brain, and their hyperactivation is implicated in late stages of neurodegenerative diseases [92].

Calpain-1 is overexpressed in the late stages of Alzheimer’s, generating toxic fragments of tau in response to Aβ aggregate treatment. Calpain-2, on the other hand, was found to show increased early activity in the pathogenesis of Alzheimer’s in a mouse model and was correlated with decreased cognitive function and increased Aβ in neocortical tissue samples from Alzheimer’s patients [92,93]. Mice with induced Machado–Joseph Disease (MJD) phenology presented an overactivated calpain system baseline and led to increased cell death in the cerebellum. Elimination of calpain-2 in mice with induced MJD phenology resulted in reduced neurotoxicity and increased survival of the mice [94]. Calpain inhibitors are known to have neuroprotective effects; therefore, pharmaceutical companies developed calpain inhibitors as potential therapeutic drugs for Alzheimer’s, among other NDs [95].

Calpain inhibition effects contributed to the neuroprotective effects exhibited by the PSO-nanoformulation commercialized as the product GranaGard^®^. The formulation contains high levels of punicic acid and resulted in the detention of Creutzfeldt–Jakob disease (CJD) for 60–80 days, followed by slower disease progression [88]. This same formulation was found to reduce Aβ formation, cyclin-dependent kinase 5 (cdk5) accumulation, and the key mitochondrial enzyme Cytochrome c oxidase in transgenic mice [43]. Additionally, ducking studies confirmed that punicic acid’s metabolite, CLA, inhibits the active site of μ-calpain, exerting neuroprotective effects against H_2_O_2_ and induced Aβ degradation in human neuroblastoma cell lines [96].

#### 3.2.3. Punicic Acid Induced a Higher Expression of GLUT4

Another common occurrence for several neurodegenerative diseases is a disturbance in glucose metabolism and the function and expression of glucose transporters. For example, hypometabolism of glucose due to a decrease in expression of glucose transporters in the brain occurs in Alzheimer’s disease [97]. Similarly, energy and glucose metabolism disturbances are suggested to play a role in the development of Huntington’s disease pathology [98]. The human brain expresses ten different sodium-independent glucose transporters (GLUTs), which in conjunction with sodium-dependent glucose cotransporters (SGLTs) and uniporter SWEET protein, are responsible for glucose uptake. GLUT4 is an insulin-sensitive glucose transporter expressed in the hypothalamus, sensorimotor cortex, cerebellum, hippocampus, and pituitary. Its physiological role is unknown, but some of its suggested functions are its involvement in glucose sensing, the insulin modulation of glucose transport in distinct brain areas, and the transport of glucose, in case of high demand, to the motor neurons [97,98].

In Alzheimer’s, along with decreased glucose uptake in highly active areas of the brain such as the cortex, hippocampus, and cerebral microvessels, glucose transporters (GLUT) decrease [98,99]. Impaired expression of GLUT-4 in the hippocampal neurons could be related to short-term memory loss and disorientation in Alzheimer patients [100]. Supplementation with three daily capsules of PSO in 52 obese patients with type 2 diabetes showed an increase in the expression of the GLUT-4 gene and a decrease in fasting blood sugar [85]. Likewise, an increase in mRNA and protein expression of GLUT4 was observed in 3T3-L1 adipocytes treated with punicic acid [83].

#### 3.2.4. Effect of Punicic Acid over HDL and PON1

Another mechanism related to oxidative stress-related diseases is the alteration of paraoxonase 1 (PON1) in circulatory plasma. The paraoxonase (PON) family of enzymes is a group of polymorphic lactonases with broad substrate specificity that have potent antioxidant, anti-inflammatory, and anti-apoptotic properties. They are highly found in HDLs, and PON1 associated with HDL helps prevent LDL oxidation [101,102]. Low levels of PON1 and HDL cholesterol are associated with a high vulnerability to oxidative damage of lipids, proteins, and DNA and elevated immune-inflammatory response. Decreased PON1 content is also related to the neurotoxic effects of the immune-inflammatory and nitro-oxidative pathways in people suffering from neuroprogressive disorders such as major depressive disorder, bipolar disorder, and schizophrenia [103]. In NDs, alterations to circulatory plasma PON1 were reported [101]. Additionally, reduction in PON1 levels is common in PD patients compared to healthy people [104].

Pomegranate induces modifications of high-density lipoproteins (HDL) lipid composition and functionality. Rabbits were supplemented during 30 days with microencapsulated pomegranate, which induced an increase in HDL cholesterol and HDL phospholipids, decreased non−HDL sphingomyelin levels, and lowered the content of the triglycerides-to-phospholipids ratio. There was an increase in HDL functionality and improved oxidation resistance, most likely as a result of reduced triglyceride levels of the HDL and an increase in PON1 activity [86]. In a similar study, women with acute coronary syndrome were supplemented with microencapsulated pomegranate for 30 days, which shifted the distribution from large HDL to intermediate and small-sized particles, and a decrease in triglyceride values and an increase in PON1 activity was observed. HDL remodeling did not change the affinity of lipoprotein for PON1 since PON1 activity remained constant before or after supplementation. This means that the higher PON1 activity after pomegranate supplementation is due to its higher synthesis [87]. Additionally, CLA isomers, particularly c9,t11, help protect PON1 from oxidative oxidation and stabilization in a concentration-dependent manner by binding to a specific binding site on a PON1 molecule [102]. Because microencapsulated pomegranate is composed of many beneficial nutraceutical components, including punicic acid, new studies need to be conducted to explore the direct effect of punicic acid over PON1 and HDL.

**Figure 6:**
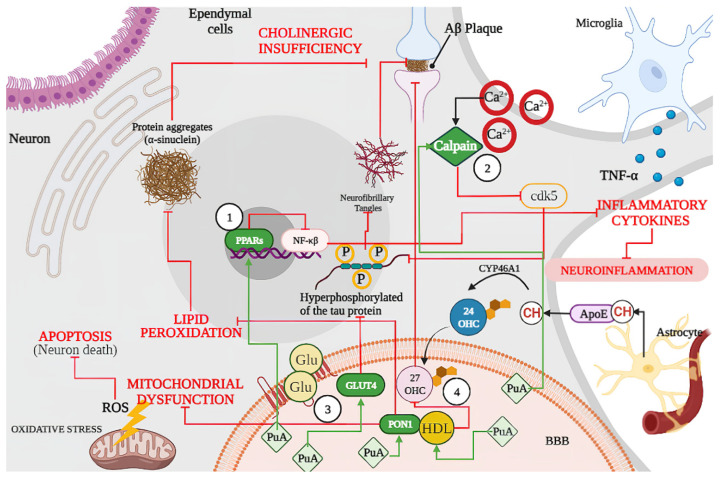
Schematic representation of biological effects of punicic acid (PuA) in neurological diseases (NDs). Punicic acid (PuA) acts as (1) an agonist of PPARs inhibiting the activation of nuclear factor kappa B (NF-κB) and the release of inflammatory cytokines such as TNF-alpha, and therefore, reducing neuroinflammation and tau hyperphosphorylation and conducting less Aβ formation and aggregation. (2) PuA inhibits activation of calpain and cyclin-dependent kinase 5 (cdk5), limiting the hyperphosphorylation of tau protein and conducting to less Aβ formation and aggregation. (3) PuA increases GLUT4 protein expression regulating the glucose brain metabolism, reducing insulin resistance, and reducing the hyperphosphorylation of tau proteins. (4) PuA increased the anti-oxidative properties of the PON1 complex reducing ROS generation limiting mitochondrial dysfunction and neuronal apoptosis. Lipids peroxidation. Moreover, PuA induces changes in high-density lipoproteins (HDL) lipid composition and functionality reducing the formation of oxysterols such as 27-hydroxycholesterol (27-OHC) and increasing oxidative resistance with less Aβ plaque formation. ROS: reactive oxygen species; PON1: paraoxonase 1; PPARs: peroxisome proliferator-activated receptors; HDL: high-density lipoprotein; GLUT4: insulin-sensitive glucose transporter; CH: cholesterol; BBB: blood–brain barrier; ApoE: apolipoprotein E; Glu: glucose, PuA: punicic acid. Green lines indicate stimulation, while red lines indicate inhibition.

In summary, punicic acid (PuA) can act as (1) an agonist of PPARs, which reduces neuroinflammation and tau hyperphosphorylation and conducts less Aβ formation and aggregation. Punicic acid reduces the Aβ formation by (2) inhibiting activation of calpain and cyclin-dependent kinase 5 (cdk5), limiting the hyperphosphorylation of tau protein. Likewise, (3) PuA increases GLUT4 protein expression regulating the glucose brain metabolism, reducing insulin resistance, and reducing the hyperphosphorylation of tau proteins. As a part of its strong antioxidant effects, (4) PuA increased the anti-oxidative properties of HDL and PON1 activity, reducing ROS generation and lipids peroxidation (Figure 6).

## 4. Concluding Remarks and Future Perspectives

Punicic acid is an important nutraceutical compound in the prevention and treatment of neurodegenerative diseases such as Alzheimer’s, Parkinson’s, and Huntington’s disease. Punicic acid can decrease oxidative damage and inflammation by increasing the expression of peroxisome proliferator-activated receptors. In addition, it can reduce beta-amyloid deposits formation and tau hyperphosphorylation by increasing the expression of GLUT4 protein and the inhibition of calpain hyperactivation. Microencapsulated pomegranate, with high levels of punicic acid, increases PON1 antioxidant activity in HDL. Likewise, encapsulated pomegranate formulations with high levels of punicic acid have shown an increase in PON1 antioxidant activity in HDL. However, punicic acid shows very low permeability across the blood–brain barrier, resulting in very limited effects on neurological disorders. In order to overcome this challenge, brain-targeted formulations that bypass the BBB have better results at diminishing ND’s symptoms, such as decreased amyloid precursor protein gene expression, oxidative stress, and neuroinflammation. Future studies that focus on the effect of punicic acid on neurodegeneration need to be mindful of the effect of the BBB on the brain bioavailability of the bioactive molecule and attempt to develop specific delivery mechanisms that allow exerting localized effects.

## Figures and Tables

**Table 1:** Effects of punicic acid over different molecules related to neurodegenerative diseases (NDs).

Molecules	Related NDs	Formulation	Effects	Mechanism	Biological Model	References
PPARγ/α and TNF-α	Alzheimer, Parkinson and Huntington, CNS Hypoxia/Ischemia	Nanoemulsified PSO supplementation	Anti-inflammationIncreased fatty acid oxidation	Gene expression upregulation of PPARγ/α/β among others	Liver of high-fat diet-fed mice.	[12]
		PuA	Improved glucose homeostasis and suppressed inflammation	Suppressed NF-κB activation and TNF-α expression via PuA Agonist effect of PPARγ	3T3-L1 pre-adipocytes and obese/high-fat diet mice	[83,84]
Calpain	Alzheimer, Parkinson and Huntington’s diseases, Machado–Joseph disease, genetic Creutzfeldt–Jakob disease	PSO-nanoformulation(GranaGard)	Detention of the disease for 60–80 days and slower disease progression after. Decreased Aβ and p25 formation.	μ-calpain inhibition and nanoformulation antioxidant effect.	Mice	[43,88]
GLUT4	Neurodegeneration	PSO	Decreased fasting blood sugar levels.	GLUT4 increased expression	Diabetic type II patients	[85]
HDL and PON1	Alzheimer, Multiple Sclerosis, Parkinson, Huntington.	Microencapsulated pomegranate	Reduction in non-HDL sphingomyelinIncrease in HDL-cholesterol and HDL-phospholipidsIncrement in PON1 activity	Reduction in triglyceride content in HDL	Rabbits	[86]
		Microencapsulated pomegranate	Decreased triglyceridesIncreased PON1 activity	Higher synthesis of PON1 protein.	Woman with Acute Coronary Syndrome	[87]

PuA: Punicic acid; PSO: Punicic seed oil; PPAR: Peroxisome Proliferator-Activated Receptor α/β/γ, TNF-α: Tumor Necrosis Factor α, GLUT4: Glucose Transporter Type 4, PON1: Paraoxonase 1, NF-κB: Nuclear Factor Kappa Beta, Aβ: amyloid-β, HDL: High-Density Lipoprotein.
